# Structural and sequence diversity of the transposon *Galileo* in the *Drosophila willistoni* genome

**DOI:** 10.1186/1471-2164-15-792

**Published:** 2014-09-13

**Authors:** Juliana W Gonçalves, Victor Hugo Valiati, Alejandra Delprat, Vera L S Valente, Alfredo Ruiz

**Affiliations:** Programa de Pós-Graduação em Genética e Biologia Molecular, Departamento de Genética, Universidade Federal do Rio Grande do Sul (UFRGS), Porto Alegre, Rio Grande do Sul CP 15053, 91501-970 Brazil; Programa de Pós-Graduação em Biologia: Diversidade e Manejo de Vida Silvestre, Universidade do Vale do Rio dos Sinos (UNISINOS), São Leopoldo, Rio Grande do Sul CP 275 93022-000 Brazil; Departament de Genètica i de Microbiologia, Universitat Autònoma de Barcelona (UAB), 08193 Bellaterra, Barcelona, Spain

**Keywords:** Transposable element, *D. willistoni*, Terminal inverted repeats, *P superfamily*, Target site duplications

## Abstract

**Background:**

*Galileo* is one of three members of the *P superfamily* of DNA transposons. It was originally discovered in *Drosophila buzzatii*, in which three segregating chromosomal inversions were shown to have been generated by ectopic recombination between *Galileo* copies. Subsequently, *Galileo* was identified in six of 12 sequenced *Drosophila* genomes, indicating its widespread distribution within this genus. *Galileo* is strikingly abundant in *Drosophila willistoni*, a neotropical species that is highly polymorphic for chromosomal inversions, suggesting a role for this transposon in the evolution of its genome.

**Results:**

We carried out a detailed characterization of all *Galileo* copies present in the *D. willistoni* genome. A total of 191 copies, including 133 with two terminal inverted repeats (TIRs), were classified according to structure in six groups. The TIRs exhibited remarkable variation in their length and structure compared to the most complete copy. Three copies showed extended TIRs due to internal tandem repeats, the insertion of other transposable elements (TEs), or the incorporation of non-TIR sequences into the TIRs. Phylogenetic analyses of the transposase (TPase)-encoding and TIR segments yielded two divergent clades, which we termed *Galileo* subfamilies V and W. Target-site duplications (TSDs) in *D. willistoni Galileo* copies were 7- or 8-bp in length, with the consensus sequence GTATTAC. Analysis of the region around the TSDs revealed a target site motif (TSM) with a 15-bp palindrome that may give rise to a stem-loop secondary structure.

**Conclusions:**

There is a remarkable abundance and diversity of *Galileo* copies in the *D. willistoni* genome, although no functional copies were found. The TIRs in particular have a dynamic structure and extend in different ways, but their ends (required for transposition) are more conserved than the rest of the element. The *D. willistoni* genome harbors two *Galileo* subfamilies (V and W) that diverged ~9 million years ago and may have descended from an ancestral element in the genome. *Galileo* shows a significant insertion preference for a 15-bp palindromic TSM.

**Electronic supplementary material:**

The online version of this article (doi:10.1186/1471-2164-15-792) contains supplementary material, which is available to authorized users.

## Background

Transposable elements (TEs) are part of the middle repetitive portion of DNA that is able to move and replicate within the genome. They comprise a considerable fraction of many eukaryotic genomes and their sequences exhibit broad structural diversity. The wide range of transposition strategies adopted by TEs involve either RNA (class 1 or retrotransposons) or DNA (class 2 or DNA transposons) intermediates. Selfish and thus in many respects indistinguishable in their behavior from parasites, these mobile genetic units increase in number within the genome because their rates of transposition are higher than those of spontaneous deletion. This evolutionary success of TEs is a major force shaping the genes and genomes of almost all organisms [1, 2].

The movement and accumulation of TEs serves as a rich source of genetic material, with a strong impact on the evolutionary reorganization of the genomes of their bearers. However, it is now clear that inactive TEs also play a significant role in macroevolution, because the most influential contributions can arise and persist long after transposition activity has ceased, such that they are manifested as TE by-products. The selfish and parasitic characteristics of TEs ensure their long-standing residence within the host genome and imply their intimate co-evolutionary relationship with it [3].

The specific features of DNA transposons compared to other TEs enhance their influence in shaping eukaryotic genomes, including the capacity to excise imprecisely, jump locally, cause multiple double-strand breaks, and undergo alternative transposition [2]. The transposon *Galileo* was originally discovered in *Drosophila buzzatii*, in wich three segregating chromosomal inversions were shown to have been generated by ectopic recombination between *Galileo* copies [4–6]. Although *Galileo* has long terminal inverted repeats (TIRs) similar to those of *Foldback-like* elements, it is classified as a member of the *P superfamily* of DNA transposons (class II, subclass 1, TIR elements order) based on the sequence of its putative transposase (TPase). Subsequently, *Galileo* was identified in six of the 12 sequenced *Drosophila* genomes of the two subgenera of *Sophophora* and *Drosophila*, indicating its widespread distribution within this genus. Although potentially active *Galileo* copies have not been found, non-autonomous copies are abundant in all species investigated [7]. In addition, two or more *Galileo* subfamilies coexisting within the same genome have been found in several cases: three subfamilies are present in *D. buzzatii* (G, K, and N for *Galileo*, *Kepler* and *Newton*), two in *D. virilis* (A and B), and five in *D. mojavensis* (C, D, E, F, and X) [6–8].

According to *in silico* predictions, *Galileo* is strikingly abundant in *Drosophila willistoni* [7], the most widespread neotropical species of the genus *Drosophila* [9, 10], with an extensive gene arrangement polymorphism on all chromosomes [11–20]. This high intraspecific polymorphism for chromosomal inversions and *Galileo* abundance suggest a role for *Galileo* in the generation of inversions in *D. willistoni* and related species. We have an ongoing project to test this hypothesis by identifying and isolating the breakpoints of *D. willistoni* natural polymorphic inversions. As a first step in this in this project, we carried out an exhaustive search for and characterization of the *Galileo* copies present in the *D. willistoni* genome. A careful and detailed annotation of 191 *Galileo* sequences revealed that they vary considerably in length and structure, ranging from nearly-complete to containing only one TIR. Two *Galileo* subfamilies with a substantial nucleotide divergence were found by phylogenetic analysis of TPase-encoding and TIR segments. In addition, by analyzing the preferred target sequence of *Galileo* in *D. willistoni*, we identified a palindromic target site motif (TSM).

## Results

### Characterization of *Galileo*copies in the *D. willistoni*genome

**Figure 1:**
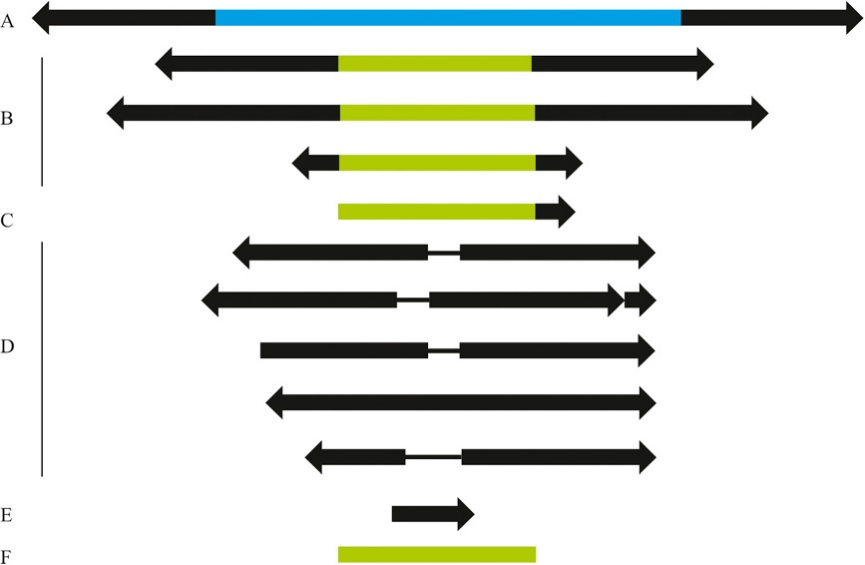
***Galileo*** **copies identified in the** ***Drosophila willistoni*** **genome were classified into the following six groups according to structure: A) Nearly-complete with two terminal inverted repeats (TIRs) and nearly-complete transposase (TPase)-encoding segment (GenBank: BK006360.1); B) two TIRs and a TPase segment; C) one TIR and a TPase segment; D) two TIRs; E) one TIR; and F) a TPase segment.** The black arrows represent the TIRs. The blue middle region in A represents the nearly-complete TPase-encoding segment. The green middle region **(B, C**, and **F**) represents a partial TPase-encoding segment. The black lines in D indicate the spacing sequences between the 5′ and 3′ TIRs. These sequences do not show homology at the nucleotide level to any known sequence in the databases.

We characterized 191 *Galileo* copies in the *D. willistoni* genome (details are given in Additional file 1), classifying them into six groups according to their structure (Table 1 and Figure 1): (A) nearly-complete; (B) two TIRs and a partial TPase-encoding segment; (C) one TIR and a partial TPase-encoding segment; (D) two TIRs; (E) one TIR only; and (F) a TPase-encoding segment. Only one nearly-complete copy, containing two TIRs and a nearly-complete TPase-encoding segment, was found. This copy, identified in previous work (GenBank: BK006360.1) [7], is 4386-bp long and harbors a long ORF (coordinates 984–3698) encoding a 905-amino-acid TPase. The only mismatch is in the start codon, with ACG = Thr instead of the canonical ATG = Met; thus, this copy cannot be functional. Nonetheless, this putative TPase is similar in size and composition to other *Galileo* elements [7]. Protein functional analysis, performed using InterProScan 4 [21], revealed the presence of a THAP domain (PF05485) in residues 14–93 (2E–12) and a THAP-domain containing a protein 9 domain (PTHR10725) in residues 251–884 (1E–61). THAP is a DNA-binding domain present in TPases of the *P superfamily*; this domain includes a Zn-coordinating C2CH signature and four other invariant residues (P, W, F, and P) that are also required for DNA binding [8]. These eight residues are fully conserved at positions C16, C21, P40, W49, C67, H70, F71, and P87 of the putative *Galileo* TPase. The second conserved domain included the triad DDE and the motif D(2)H, which is present in the catalytic domain of cut-and-paste TPases of the *P superfamily* [22] at positions D327, D415, E642, and D449(2)H452.

**Table 1:** ***Galileo*** **copies characterized in the** ***Drosophila willistoni*** **genome**

Group	Number of copies	Copies with inserted TE	Copies with flanking TE	Copies with inserted and flanking TE
A	1	0	1	0
B	7	5	0	0
C	26	11	0	3
D	124	11	19	2
E	2	0	1	1
F	31	6	7	3
Total	191	33	28	9

TE, transposable element.

Most (~89%) of the copies with preserved terminal sequences are flanked by identical target-site duplications (TSDs). Approximately 17.3% (33 copies) contain other elements inserted within them, 14.7% (28 copies) have other elements inserted at the *Galileo* termini, and 4.7% (9 copies) have both inserted and flanking elements (Table 1). In one case, we identified a full-length *P element* (99% identity with the *D. melanogaster P element*) [23], possibly with imperfect TSDs (CGCTAGCC/GGCTAGCG) inserted within the *Galileo* copy, that contained only fragments of TIRs and identical TSDs. Of the copies with a TPase-encoding segment only (group F), 58% (18 copies) are located at the ends of short scaffolds (≤5,598-bp); thus, they may be incomplete, either because the rest of the sequence is present somewhere else or it is missing. None of the copies in groups B–F have an intact ORF encoding a putatively functional TPase (i.e., all characterized copies are non-autonomous; with variable portions of the TPase-coding region).

### TIR structural variation

*Galileo* copies in the *D. willistoni* genome exhibit remarkable structural variation. In particular, the TIRs vary considerably in length and structure compared to the TIRs of the nearly-complete copy (Figure 1), which are 765/757-bp long and have 99% identity (omitting indels). The 3′ TIR has a 69-bp overlap with the TPase-coding segment (Figure 1). Thus, the final piece of this segment is repeated (in reverse orientation) at the 5’ TIR. This is a unique trait among the described *Galileo* transposons [7]. Also, there are two AT-rich segments, with the 136-bp segment located in the 5′ TIR (coordinates 528–663) and the 137-bp segment located in the 3′ TIR (coordinates 3732–3868).

**Figure 2:**
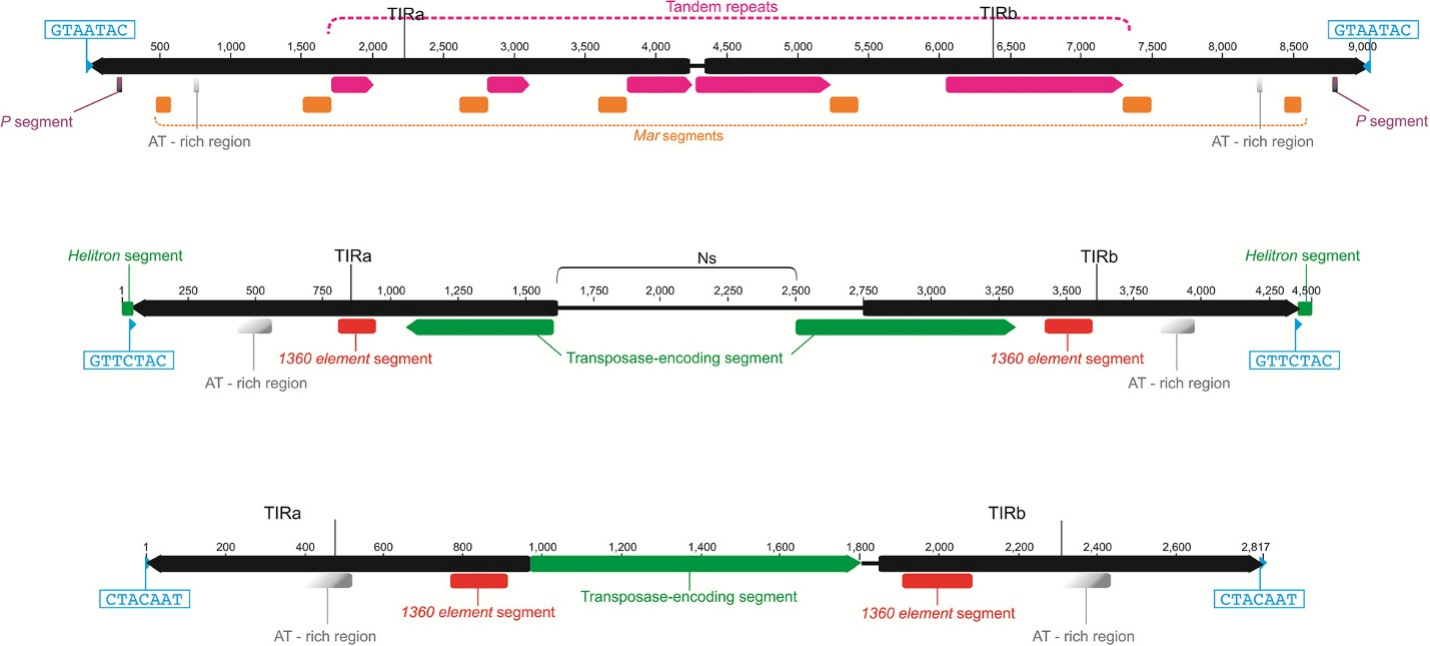
**Schematic representation of the** ***Galileo*** **copies with the longest TIRs (copies 112, 147, and 145).** The black and pink arrows represent the TIRs and the direct tandem repeats, respectively. Transposable element (TE) insertions are shown as solid rectangles: orange (*Mar*), purple (*P*), green (*Helitron*), red (*1360*). Gray rectangles represent A-T-rich regions. The green middle arrows indicate the TPase-encoding segment. In the second copy (147). The gap between the TIRs (filled with Ns) is indicated by a black line. Small blue arrowheads represent the TSDs.

**Figure 3:**
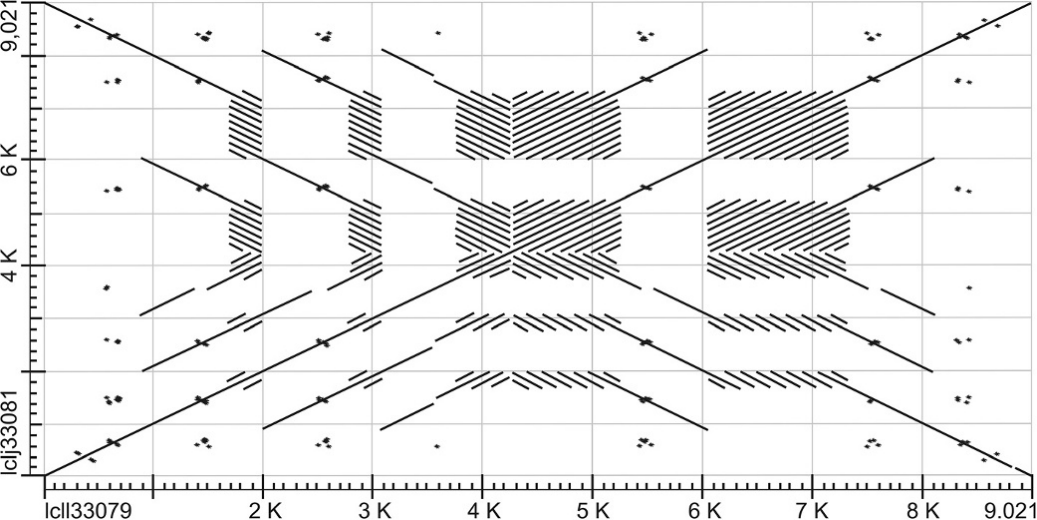
**Dot-matrix plot of the longest** ***Galileo*** **copy in the** ***D. willistoni*** **genome.** The copy structure contains two long TIRs. The principal diagonal line with disruptions (insertions and deletions) shows the alignment between the two TIRs. Additional repetitive regions are represented by diagonally striped rectangles.

Three *Galileo* copies were found to display significantly extended TIRs and each one is flanked by identical TSDs (Figure 2). The longest copy (112) is 9021-bp long, including TIRs of 4246-bp and 4680-bp (5′ and 3′, respectively; see Additional file 1). This copy contains only two TIRs and it lacks a TPase-coding segment. However, the TIRs are notable for their striking length and repetitive structure (Figure 3). They contain direct tandem repeats and an insertion of another TE in addition to the AT-rich segments. This longest copy is the only one with direct tandem repeats within the TIRs. The repeats are ~140-bp long and located approximately 1710-bp and 1730-bp from the 5′ and 3′ ends, respectively. The 5′ TIR contains three repeat regions, two that are 275-bp long (2 tandem repeats) and another that is 443-bp long (3.2 repeats). On the 3′ end, we annotated two longer repeat regions, a 995-bp region (6.8 repeats) and a 1246-bp region (9 repeats) (Figures 2 and 3). The TIRs of this copy contain fragments of two additional transposons: *P element* and *Mar.* At the 5′ and 3′ ends, we identified one fragment (36-bp) of a *P element* (86.1% identity with the *D. bifasciata P element*, according to the database; coordinates: 2900–2935) [24]. Of the seven fragments of *Mar* that were annotated, two are 107-bp long (one at each end) and the other five are 202-bp long (98.1% and 94.5% identity with *D. willistoni Mar*, respectively; coordinates 491–597 and 299–500) [25].

The copy (147) with the second longest TIRs (1575-bp and 1608-bp; 97.5% identical) is 4306-bp long (Figure 2). The TIRs are composed of: 130-bp of AT-rich sequence, 140-bp and 172-bp stretches (5′ and 3′ TIR, respectively) similar to transposon *1360* (also known as *Hoppel* or *ProtoP* element; 85.7% identity with coordinates 4020–3869 and 84.9% identity with coordinates 3869–4079, respectively) [26, 27], and at least 545-bp of the *Galileo* TPase (coordinates: 3700–3162). Thus, in this *Galileo* copy, TPase-encoding segments are repeated, forming part of the TIRs (Figure 2). In addition, there is a 872-bp gap between the TIRs (filled with Ns) that may hide a larger TPase fragment. The third copy (145) has TIRs that are 959-bp (99.9% identical) in length, with 132-bp of AT-rich sequence and the same fragments of the*1360* transposon present in the copy previously described. These last two copies are similar in their structure and have 99.8% identity over the first 959-bp. However, they have different TSDs, indicating that they are independent insertions.

### *Galileo*sequence diversity in the *D. willistoni*genome

**Figure 4:**
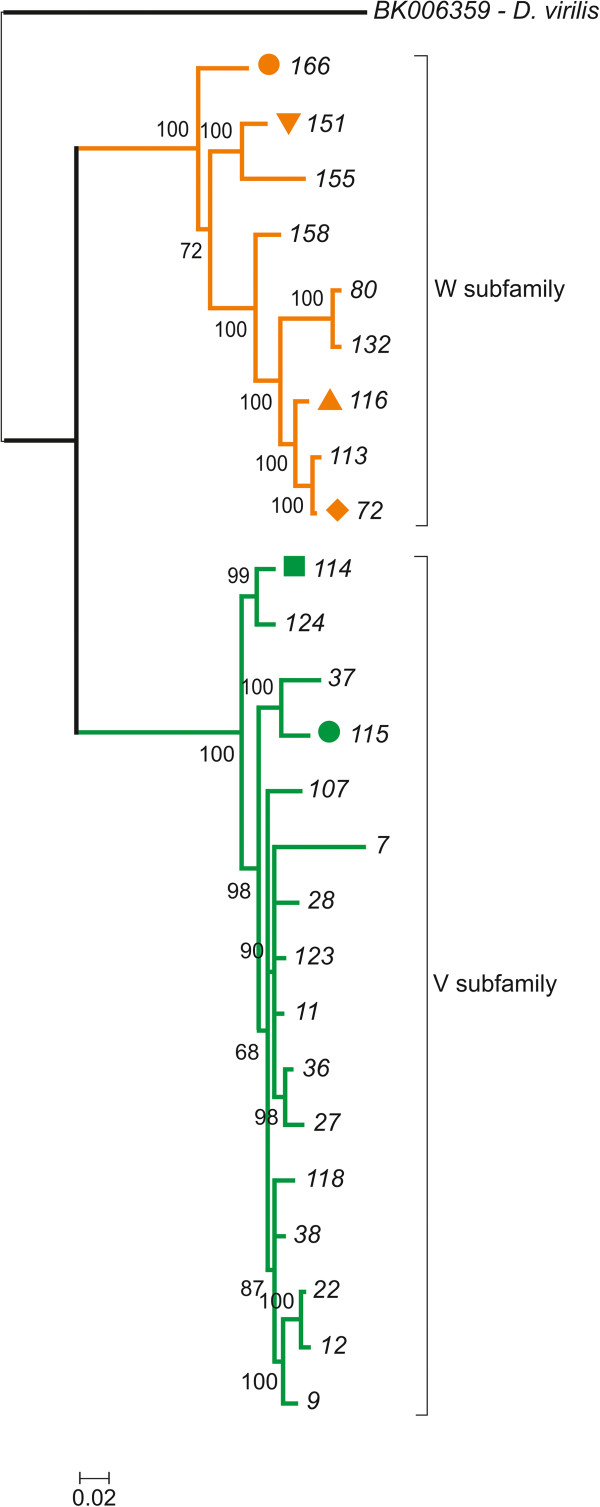
**Bayesian inference tree constructed with** ***Galileo*** **TPase-encoding segments.** The bootstrap values of each group node are indicated (values below 50% were omitted). The two subfamilies, V and W, are strongly supported. Copies highlighted with symbols are also present in the phylogenetic analysis of the TIRs. *Galileo* (nearly-complete copy) of *D. virilis* was used as the outgroup.

We aligned the TPase-coding segments from 26 *Galileo* copies and used a ~488-bp region (coordinates: 2699–3131) to build a phylogenetic tree using maximum-likelihood (ML) and Bayesian inference (BI) methods. The phylogenetic trees of the TPase-coding sequences created using the two methods were similar and recovered the same two clades with significant statistical support (Figure 4). The two clades showed a substantial nucleotide divergence between them (20%–50.3%) and were termed *Galileo* subfamilies V and W. The analysis placed the nearly-complete copy within the W subfamily. Assuming a *Drosophila* synonymous substitution rate of 0.016 substitutions per nucleotide/myr [28], we estimated the split between the two subfamilies to ~ 9 million years ago (Mya). The two subfamilies have a modest mean divergence between copies within each one (4.7 and 6.7%, respectively) compared with the mean divergence between them (24%) (Table 2).

**Table 2:** **Nucleotide divergence estimates (%) for the** ***Galileo*** **subfamilies using transposase-coding sequences**

	V subfamily	W subfamily	***Galileo***of ***D. virilis***
V subfamily	4.7 ± 0.60		
W subfamily	24.02 ± 2.19	6.66 ± 0.086	
*Galileo* of *D. virilis*	32.34 ± 2.52	30.08 ± 2.60	-

*Galileo* (nearly-complete copy) of *D. virilis* was used.

**Figure 5:**
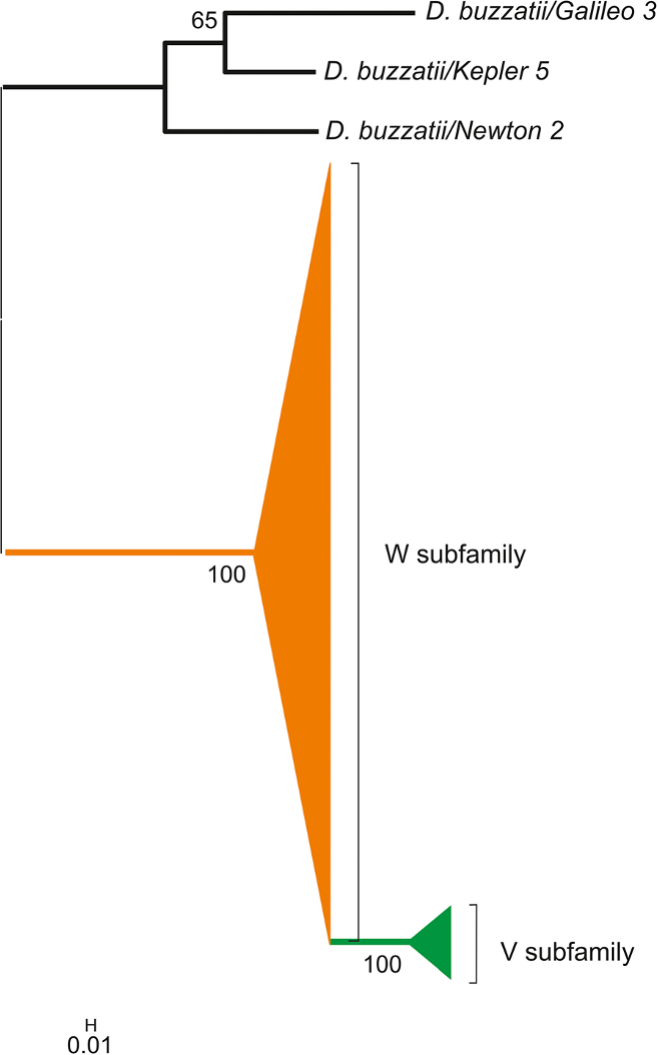
**Bayesian inference tree showing the relationships among** ***Galileo*** **TIRs.** The groups were compressed to facilitate visualization; therefore, 114 and 24 sequences were omitted from the W and V subfamilies, respectively. The bootstrap values of each group node are indicated (values below 50% were omitted). The two subfamilies, V and W, are strongly supported. The *Galileo*, *Kepler*, and *Newton* subfamilies of *D. buzzatii* were used as the outgroup in the phylogenetic analysis.

We also aligned the TIR regions and built a phylogenetic tree with 238 homologous segments (the first 100-bp). The topologies of the TIR trees also yielded two clades, with 11%–26.6% nucleotide divergence (Figure 5). Several copies (72, 114, 115, 116, 151), in addition to the nearly-complete copy (166), contain both the initial portion of the TIR and a TPase-encoding fragment. The phylogenetic placement of these copies suggests that the two clades in the TIR phylogeny correspond to the above-defined V and W subfamilies (Figure 5 and Additional file 2). Subfamily V was represented by copies with extended TIRs (145 and 147) that have the homologous TIR region (the first 100-bp). There was no significant difference in the lengths of the TIRs among subfamilies. The mean divergence between copies of the two subfamilies was 13.6%, whereas the mean divergence within subfamilies was much smaller (1.3% and 3.1%) (Table 3). The divergence within subfamilies includes the estimates between copies and between the two TIRs within copies.

**Table 3:** **Nucleotide divergence estimates (%) for the** ***Galileo*** **subfamilies using the terminal inverted repeat regions**

	V subfamily	W subfamily	Subfamilies of ***D. buzzatii***
V subfamily	3.08 ± 0.09		
W subfamily	13.61 ± 3.57	1.31 ± 0.024	
Subfamilies of *D. buzzatii*	52.92 ± 3.83	48.51 ± 4.91	28.41 ± 4.02

*Galileo* subfamilies K, G and N of *D. buzzatii* were used.

**Figure 6:**
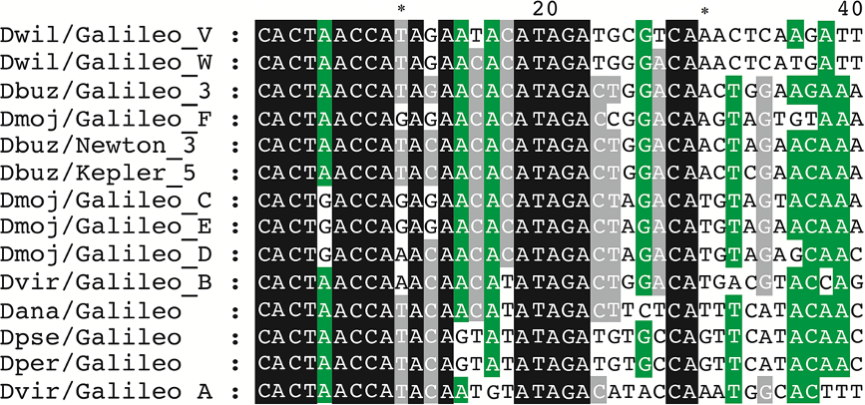
**Comparison of the TIRs ends.** A consensus sequence was constructed for the V and W subfamilies of the *Galileo* TIRs in *D. willistoni*. Alignments of the 40-bp TIRs of each *Galileo* subfamily and species are shown. Identical positions (17) in all sequences are marked in black, and the 80% and 60% conserved positions in green and gray, respectively.

The consensus sequences for the terminal 40-bp segment in *Galileo* subfamilies V and W differed by 4 bp (10%). A comparison of the 40 terminal bp region conserved in 14 *Galileo* sequences of diverse species and subfamilies showed a total of 17 conserved nucleotides (Figure 6).

### Target site duplication and target site motif

**Figure 7:**
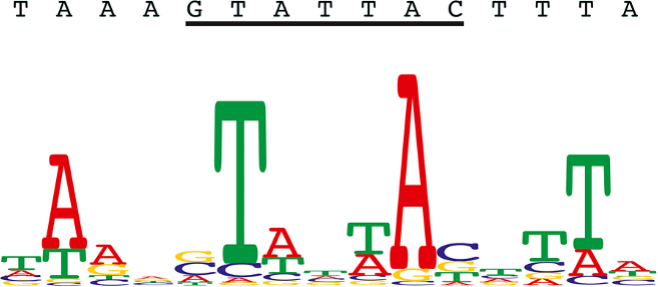
**Sequence logo and consensus sequence for the 7-bp TSD and the 15-bp target site motif.** The sequence underlined in black is the target preference for *Galileo* in the *D. willistoni* genome*.*

In most *D. willistoni Galileo* copies, the TSDs were 7-bp in length, as similarly reported in *D. buzzatti* [29]. However, we identified three copies (89, 127, and 179) in which the TSDs were 8-bp long (see Additional file 1). Comparison of the 118 flanking sequences of those *Galileo* copies with the 7-bp TSD suggested that the consensus sequence of their preferential insertion site is GTATTAC (Figure 7).

The majority-rule consensus sequence for the flanking sequences of *D. willistoni Galileo* copies suggested that the insertion sites are localized at the center of the AT-rich region. Analysis of the 93-bp surrounding the TSDs revealed a target site motif (TSM) with a 15-bp palindromic pattern composed of 7-bp duplicated upon insertion plus 4-bp on either side of *Galileo* (Figure 7). Two pentanucleotides, AANGT and ACNTT, were identified on the 5′ and 3′ halves of the TSM [30]. This motif could adopt a stem-loop secondary structure when denatured.

## Discussion

In the present study, we used different search strategies and a detailed manual annotation to fully characterize *Galileo* copies in the *Drosophila willistoni* genome. In contrast to previous work [7], which reported on 28 copies, this study presents information on 191 copies. The long term goal of this project is to contrast the hypothesis that *Galileo* generated some of the *D. willistoni* chromosomal inversions segregating in natural populations. The detailed annotation of all *Galileo* copies present in the *D. willistoni* genome will greatly assist in the interpretation of the breakpoint sequences.

### *Galileo*structural variation

Putatively functional copies of *Galileo* were not found, although one nearly complete copy harbors an ORF coding for a 905-amino-acid TPase (after curating a mismatch in the start codon). Among the non-autonomous copies with TPase segments, the majority (~63.6%) were composed of TIRs and a spacing region. In addition, they exhibit a remarkable structural variation, particularly in the TIRs. *Galileo,* along with two other transposons, *P‒element* and *1360*, are members of the *P superfamily* [31]. *P* elements move to a new site through a non-replicative process, i.e., the cut-and-paste mechanism of transposition, in which the excised copy leaves behind a double-strand gap [32]. Because gap repair is not always efficient, whether via homologous recombination or using the sister chromatid strand as a template, defective copies are often generated due to abortion, slippage, or template switching in the course of transposition and repair [2, 33]. Furthermore, because transposons are dispersed repeats in the genome, non-allelic homologous recombination or ectopic recombination events are likely, thereby increasing the probability of exchange between two copies and affecting the structure of the sequences. These molecular processes can explain the gradient of *Galileo* copies found in the *D. willistoni* genome, ranging from an almost-complete copy to defective copies restricted to the TIRs, with various degrees of degeneration.

Moreover, *Galileo* displays dynamic restructuring. A recent analysis of the *Drosophila mojavensis* genome [8] identified two patterns of extension for *Galileo* TIRs: (1) expansion of the direct tandem repeats and (2) recruitment of internal sequences (non-TIR segments) into the TIRs. In the *D. willistoni* genome, we identified direct tandem repeats within the TIRs, but in a single copy only (the longest one, Figure 2). We also found evidence of recruitment of non-TIR segments into the TIRs. Remarkably, in the nearly-complete copy, a 69-bp final piece of the ORF is now part of the 3′ TIR, and it is repeated (in reverse orientation) at the end of the 5′ TIR. In other copies, the overlap between the ORF and TIRs is even greater. For instance, in copy 147, the segment of the ORF recruited to the TIRs is more than 500-bp long (Figure 2). This is, so far, a unique trait of *Galileo* transposons [7]. Finally, we found a third pattern of TIR extension: the insertion of another TE into one of the TIRs, which eventually may be transferred to the other TIR, ultimately becoming a part of both. The TE fragments are not occasional insertions in one *Galileo* TIR; rather they are part of the 5′ and 3′ ends in transposing copies. We detected the insertion of three elements in the longest TIRs, i.e., *P*, *Mar*, and *1360*. The first two were previously studied in *D. willistoni* [34–36], but the origin of the *1360* fragment is obscure because in *D. willistoni* transposon *1360* is missing [7].

### Two *Galileo*subfamilies in the *D. willistoni*genome

In previous work [7], a limited number of *Galileo* copies were isolated from the *D. willistoni* genome, and a subsequent phylogenetic analysis did not detect a significant structure. Here, our phylogenetic analysis, based on an increased number of copies, revealed two strongly supported clades, which we named subfamilies V and W. The two clades were evident in phylogenetic analyses carried out using either a segment of the ORF or the final 100-bp of the TIRs. Although in our study only six copies were shared between the two phylogenies, two in subfamily V (114, 115) and four in subfamily W (72, 116, 151 and 166), the results are congruent and suggest the same grouping in the two subfamilies. The presence of *Galileo* subfamilies within the same genome seems to be the rule rather than the exception, as was previously found in *D. buzzatii* (subfamilies G, K, and N), *D. virilis* (subfamilies A and B), and *D. mojavensis* (subfamilies C, D, E, F, and X) [6–8]. Furthermore, the coexistence of different subfamilies, subgroups, or variants of TEs was reported in studies of *Bari* [37] and G*ypsy* [38] in *Drosophila*, *P* in *Anopheles gambiae* and *Drosophila* [39, 40], and *mariner* in insects and humans [41, 42], among others.

How have these *Galileo* copies differentiated in the genome of *D. willistoni*? Horizontal transfer (HT) and vertical diversification are the two main hypotheses that explain the coexistence of different subfamilies in the same genome [2]. HT would account for the appearance of the two subfamilies, via two independent events of *Galileo* invasion in the *D. willistoni* genome. Several mechanisms and vectors have been proposed to explain HT events. In *Drosophila* parasites and parasitoids, such as mites and wasps, intracellular symbiotic bacteria, such as *Wolbachia* and spiroplasms, are possible vectors of TEs [43]. HT can also result from an introgression, as reported in the *willistoni* subgroup [44–46], and is a potential mechanism for *P* element spreading among this subgroup [47]. Although the HT hypothesis in the case of *Galileo* has yet to be disproven, our data suggest that, based on the landscape of this transposon in *D. willistoni*, the copies instead diverged from an ancestral element in the genome. Although no complete copies of *Galileo* have been found, its functional differentiation would have had to be driven by specific selective pressures, resulting in the formation of two distinct *Galileo* TPases to overcome cellular repression of transposition. We identified *Galileo* copies composed of TIRs with conserved TPase site affinity in the genome; these could have served as a source for the other defective copies. Furthermore, HT and vertical diversification are not mutually exclusive; thus, successive invasions and structural variations may have occurred during the diversification of TEs. Concerning the preservation of *Galileo* TIRs, the mean divergence for these sequences was only one half (~13.6%) of that for the TPase-encoding segment (~24%). Under a neutral evolution model, the same degree of divergence would be expected; however, in the case of *Galileo*, there are more constraints in the terminal segment (100-bp) of its TIRs than in its TPase-encoding segment because the former are required for transposition.

### *Galileo*insertional preference

DNA transposons generate, upon insertion, direct duplications of short genome sequences (TSDs). In *D. buzzatii*, a comparison of the 19 flanking sequences suggested that *Galileo* generates 7-bp TSDs with the consensus sequence GTAGTAC [48]. A larger sample (106 *Galileo* copies) in six sequenced *Drosophila* genomes (*D. ananassae*, *D. pseudoobscura*, *D. persimilis*, *D. willistoni*, *D. virilis*, and *D. mojavensis*) identified the consensus sequence GTANTAC [7]. We found that the *Galileo* TSDs in *D. willistoni* are typically 7-bp but, as occurs in most *P* element insertions, three *Galileo* copies had TSDs of 8-bp. Additionally, by comparing 118 *Galileo* copies flanked by identical 7-bp sequences, we were able to infer that the preferential insertion site has a consensus sequence of GTATTAC, in which the fourth position differs from that occurring in *Galileo* copies in *D. buzzatii*. These findings are in agreement with those of a study of six *Drosophila* genomes. Linheiro and Bergman [30] measured the degree of target specificity for different elements in *D. melanogaster*. They found that *1360* and *P* elements seem to have a relatively low degree of target specificity. *Galileo* seems to have a higher target specificity than either *1360* or the *P element*. Accordingly, it can be detected with a lower number of insertions [7, 29, 48].

A previous study identified a 14-bp palindromic pattern centered on the 8-bp TSD generated by *P* element insertion [49]. Sequence motifs at TE target sites are always palindromes that extend beyond the TSD [30]. Here, by analyzing the region around the TSDs, a 15-bp palindrome was identified; in addition, the *Galileo* TSM also had a general tendency to be AT-rich. Although the tendency in the TSMs of both *P* element and *1360* is to have an ANAGT motif in the 5′ half and an ACTNT motif in the 3′ half, the Galileo TSM while palindromic, is not identical in sequence (AANGT and ACNTT, respectively).

## Conclusions

Our detailed analysis of 191 *Galileo* copies revealed an enormous variety in their size and structure. In some copies, there were different forms of TIR extension, including internal duplications, recruitment of the final piece of the TPase-encoding ORF into the TIRs and secondary TE insertions in one TIR that subsequently become part of both TIRs. Two *Galileo* subfamilies (termed V and W) coexist in the *D. willistoni* genome. They are evident in the phylogenetic trees of both the TPase-encoding and the TIR segments. However, phylogenetic analysis showed that the divergence between and within subfamilies is smaller in the TIR segment than in the TPase-encoding segment, presumably because the former is required for transposition. *Galileo* shows a stronger target preference that *1360* or *P-element*, the other two members of the *P* superfamily.

## Methods

### Bioinformatic searches

The *D. willistoni* genome sequence was used for *in silico* analyses. Candidate *Galileo* elements were identified by querying the nearly-complete copy of *Galileo* detected in the *D. willistoni* genome in previous work [7], terminal inverted repeats (TIRs), and segments of the transposase (TPase)-encoding ORF isolated by experimental searches of *Galileo* in *D. willistoni* (Gonçalves et al. in preparation). Blast searches of the *Drosophila willistoni* genome were performed using FlyBase [50], with default parameters and without a low complexity filter, to identify copies with simple and complex repeats. The applied threshold of scores had an e-value of <10^-4^. To accept a search hit, we compared previously characterized copies and identified characteristic structures.

When a segment of the *Galileo* TPase was used as the query subject, to identify TIRs and target site duplications (TSDs), pairwise comparisons of upstream and downstream flanking sequences (up to 5000-bp, if available) were carried out. TSDs were identified by aligning 50-bp upstream and downstream of the TIRs of the TEs. The target site motif (TSM) was constructed by concatenating the flanking sequence upstream of the element insertion, containing the TSD (50-bp), and the flanking sequence (43-bp) downstream from the element insertion, lacking both this element and the TSD. Hits were considered part of the same *Galileo* copy if arranged in the proper orientation at a distance of <5 Kb.

### Annotation of the *Galileo*copies

The detected sequences were manually annotated using different tools, most of which were implemented using Geneious R6, created by Biomatters [51], and custom Blast searches using specific *Galileo* and *Drosophila* TE databases. To avoid and discard false automatic identifications, all hits from each search were manually curated. Similarity searches were used to identify and annotate the insertions of other TEs inside *Galileo* by Blast searches, carried out using the National Center for Biotechnology Information (NCBI) [52] and RepeatMasker [53] databases. Default parameters were applied, except for basic options, which we set as follows: cross-match in the search engine, slow in speed/sensitivity, and specify *Drosophila* in DNA source. To verify the presence of direct repeats, we used the Tandem Repeat Finder program, with default parameters [54]. Thus, we identified the following regions in each *Galileo* copy: TIRs, the TPase-coding region, and insertions and repeats.

### Phylogenetic analysis

Phylogenetic trees were built using the TPase-coding sequences (~630-bp) and the homologous TIR region (100-bp). The sequences were aligned with MAFT software [55]. Phylogenetic analyses were conducted with the maximum likelihood (ML) method, using PHYML 2.4.4 [56] and Bayesian inference of phylogeny (BI) using MrBayes 3.1.2 [57], applying default priors and three heated, one cold Markov chains and running each analysis from two random starting points. For TPase segments, the Akaike’s information criterion (AIC, Akaike 1974) indicated that the HKY + G model [58] was the best fit-model of sequence evolution (-lnL = 2502.9929, AIC = 5015.9858) and of the gamma-distribution shape parameter (5.7262). The Markov chain Monte Carlo search was run with 10,000,000 generations (repeated two times), with sampling conducted every 1,000 generations. The first 25% of the trees were discarded as "burn-in", at which time the chain reached stationarity, ensuring that the average split frequencies between the runs was < 1%. For the TIR sequences, the AIC indicated that the GTR model [59] with an equal gamma distribution rate was the best fit-model of sequence evolution (-lnL = 785.0532, AIC = 1586.1063). The Markov chain Monte Carlo search was run with 10,000,000 generations (repeated tree times) and sampled every 1,000 generations. The first 25% of the trees were discarded as "burn-in", at which time the chain reached stationarity. MEGA version 5.1 was used to calculate the average divergence within and between *Galileo* subfamilies, and the p-distance model and 1,000 bootstrap replications were used to date the divergence between the V and W subfamilies, calibrating the tree with the synonymous substitution rate of 0.016 substitutions per site per million years, as calculated for *Drosophila* genes with a low codon usage bias [28, 60].

### Identification of insertion sites

Insertion sites were analyzed by extracting the flanking sequences (50-bp) upstream and downstream of the element insertion, i.e., those lacking the element and TSD. For this analysis, we restricted the data to those from insertions for which the TSD sequence from each end of the element could be independently determined. To examine the potential secondary structure formed at the insertion site, we used the m-fold web server [61] to analyze the majority rule consensus sequence of the sequences around the TSDs; default parameters were applied, except in the case of the "folding temperature", which was set to 23°C.

## Electronic supplementary material

Additional file 1: ***Galileo*** **copies characterized in the** ***D. willistoni*** **genome.** The terminal inverted repeats (TIRs) positioned at the 5′ and 3′ ends are indicated as TIRa and TIRb, respectively. When the two target site duplications (TSDs) are exactly the same, only one sequence is given. Total length is expressed in base pairs for copies flanked by identical TSDs, with the insertions of other transposable elements (TEs) indicated as appropriate. The length was not estimated for fragmented copies or for those copies without preserved TSDs. Insertions with homology to known elements are indicated with the name of the corresponding TE, and their coordinates are provided according to the database and their localization in the *Galileo* copy. (XLSX 34 KB)

Additional file 2: **Bayesian inference tree showing the relationships among** ***Galileo*** **terminal inverted repeats.** The bootstrap values of each group node are indicated (values below 50% were omitted). The two subfamilies, V and W, are strongly supported. Copies highlighted with symbols are also present in the transposase-encoding segments, as determined in the phylogenetic analysis. *Galileo*, *Kepler*, and *Newton* subfamilies of *D. buzzatii* were used as outgroup in the phylogenetic analysis. (TIFF 125 KB)
