# Supplementary figures and images for: Structural and sequence diversity of the transposon Galileo in the Drosophila willistoni genome
Source: BMC Genomics. 2014 Sep 13;15(1):792. doi: 10.1186/1471-2164-15-792 (PMC4168063; doi:10.1186/1471-2164-15-792)

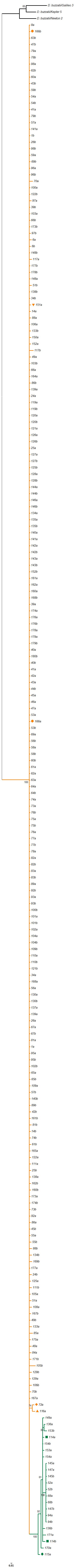

Supplement: Supplementary file 2 — Additional file 2: Bayesian inference tree showing the relationships among Galileo terminal inverted repeats. The bootstrap values of each group node are indicated (values below 50% were omitted). The two subfamilies, V and W, are strongly supported. Copies highlighted with symbols are also present in the transposase-encoding segments, as determined in the phylogenetic analysis. Galileo, Kepler, and Newton subfamilies of D. buzzatii were used as outgroup in the phylogenetic analysis. (TIFF 125 KB) [file 12864_2014_6454_MOESM2_ESM.tiff]
